# Titania Nanofluids Based on Natural Ester: Cooling and Insulation Properties Assessment

**DOI:** 10.3390/nano10040603

**Published:** 2020-03-26

**Authors:** Cristian Olmo, Cristina Méndez, Félix Ortiz, Fernando Delgado, Alfredo Ortiz

**Affiliations:** Electrical and Energy Engineering Department, University of Cantabria; Santander, 39005 Cantabria, Spain; cristina.mendez@unican.es (C.M.); felix.ortiz@unican.es (F.O.); fernando.delgado@unican.es (F.D.); alfredo.ortiz@unican.es (A.O.)

**Keywords:** cooling, experimental platform, insulation, nanofluid, transformers

## Abstract

The assessment of a TiO_2_ vegetal-based dielectric nanofluid has been carried out, and its characteristics and behavior have been tested and compared with a previously tested maghemite nanofluid. The results obtained reflect a similar affectation of the main properties, with a maximal improvement of the breakdown voltage of 33% at 0.5 kg/m^3^, keeping the thermal conductivity and the viscosity almost constant, especially the first one. This thermal characterization agrees with the results obtained when applying the TiO_2_ optimal nanofluid in the cooling of an experimental setup, with a slightly worse performance than the base fluid. Nevertheless, this performance is the opposite to that noticed with the ferrofluid, which was capable of improving the cooling of the transformer and decreasing its temperature. The similarities between the characterizations of both nanofluids, the differences in their cooling performances and their different magnetic natures seem to point out the presence of additional thermomagnetic buoyancy forces to support the improvement of the cooling.

## 1. Introduction

The continuous improvement of available equipment, technologies and work methodologies is mandatory in the field of engineering. In the case of power transformers, the engineers are always trying to enhance their efficiency and power; to reduce the amount of materials required for their construction and their size; and to make them more secure or environment-friendly by, for example, replacing the dielectric mineral oil with biodegradable alternatives. As in other scientific fields, a possible improvement of the dielectric oils could arise from nanotechnology. The use of nanoparticles is growing in importance in medicine, in detection and treatments against cancer and in the administration of medicines [1,2]; in material science, for obtaining polymers and materials with enhanced properties for several applications [3,4,5]; in the treatment of industrial wastes, as they are able to capture polluting substances [6]; and in energy and thermal engineering, for energy and heat storage and cooling [7,8,9,10]. The research on dielectric nanofluids is being carried out under this context, aiming at the improvement of the cooling and the electrical isolation of the transformer wires.

A brief review of these works reflects the theoretical suitability of the dielectric nanofluids for this purpose. From the thermal standpoint, the addition of nanoparticles (mainly metal oxides) to a dielectric base oil seems to enhance its thermal conductivity (k) or cooling capacity. This was first noticed with other cooling base fluids, including water and etilenglicol [10,11,12,13]. Regarding their expected behavior as insulators, the presence of nanoparticles in a suspension improves the dielectric strength of the base fluid, as the breakdown voltages (BDV) of the tested nanofluids are usually higher.

More precisely, Chiesa et al. [14], Choi et al. [15] and Xie et al. [16] characterized Al_2_O_3_ oil-based nanofluids of different concentrations between 0.25 and 5 vol.%. They found enhancements of the thermal conductivities of nanofluids with respect to the base fluids of between 2% and 38%, with similar results for comparable concentrations. This was not the case with SiO_2_ nanoparticles, also investigated by Chiesa [14], as the variation of the thermal conductivity was half. Additionally, with a TiO_2_ solid fraction of 0.1 vol.%, studied by Jin et al. [17] and Lv et al. [18], k variations up to 1.2% were seen. On the contrary, according to Nkurikiyimifura et al. [19], iron species have shown better improvements of the thermal conductivity, as with nanoparticle concentrations between 1 and 5 vol.%, enhancements of k in the range 10%–60% were reached. Peppas et al. [20] also studied these nanofluids with similar results, as the cooling capacity of the base fluid was improved up to 45%, but with concentrations more than one hundred times lower than those used by Nkurikiyimifura. In general, in these works, the dependence of the k enhancement on the nanofluid concentration was observed, as a larger presence of nanoparticles produces larger increases of k.

In parallel, Hanai et al. [21] and Muangpratoom et al. [22] discovered improvements of BDV of base fluids close to 25% due to the presence of 0.05 and 0.03 vol.% of TiO_2_ nanoparticles respectively. Nevertheless, this parameter started to decrease at higher concentrations, with some BDV results being under those of the base fluid, according to Hanai research. Peppas et al. [20], Irwanto et al. [23], Rafiq et al. [24] and Primo et al. [25] found a similar situation with Fe_2_O_3_ and Fe_3_O_4_ nanofluids in different concentrations (0.1 to 0.5 kg/m^3^).

In view of these tendencies, it seems necessary to reach an equilibrium of the effects on the different parameters that condition the performances of the nanofluids, which include both dielectric and thermal properties. Although the nanoparticle concentration promotes the thermal conductivity, an excess of them can harm the dielectric strength, or even the thermal convection, due to increases in viscosity [15]. Additionally, as can be noticed in the aforementioned studies, and in available reviews [26,27,28], most of the work in this field has focused on measurements of dielectric and thermal properties of mineral-based nanofluids. Therefore, starting from this available information, a closer approach to actual transformer oil operation conditions was needed.

In this sense, a study about low-concentrated maghemite (one of the most promising nanoparticles) nanofluids was carried out, whose results are published in [29]. This first study includes, among other things, a characterization of the main properties of several Fe_2_O_3_ nanofluids in a narrow range of concentrations, as well as one of the base fluid, for comparison’s sake. The results were in line with other investigations. They showed how the nanoparticles affected each of the properties measured and enabled the authors to infer an optimal concentration of nanoparticles in the base fluid based on this characterization. A novel second stage was included in this research with respect to other, similar works in transformer nanofluids. The base fluid and the optimal nanofluid were subjected to cooling performance tests in an experimental setup with a real transformer under different load conditions. An improvement of the cooling capacity was noticed, regarding the performance of the base fluid, despite the results obtained from the characterizations of the viscosity and thermal conductivity of each sample. The theoretical appearances of thermomagnetic buoyancy forces in the Fe_2_O_3_ nanofluid were given to explain this behavior, although further investigation was needed.

Among the further investigations proposed in [29], the realization of similar tests with non-magnetic nanoparticles has been carried out, emphasizing a real application of the nanofluids, at the laboratory scale. The objective was to check whether the beneficial effect of the TiO_2_ nanoparticles on the base fluid tested would be similar for those with magnetic nanoparticles. If so, the presence of a magnetic cause for the improvement of the cooling could be excluded. This paper presents the results of this experimental study on the impact of titania nanoparticles (TiO_2_) on the thermal-dielectric properties and cooling capacity of a commercial natural ester, following the same procedure exposed in the previous paper, thereby supplementing and completing the previous investigation.

## 2. Materials and Methods 

**Figure 1:**
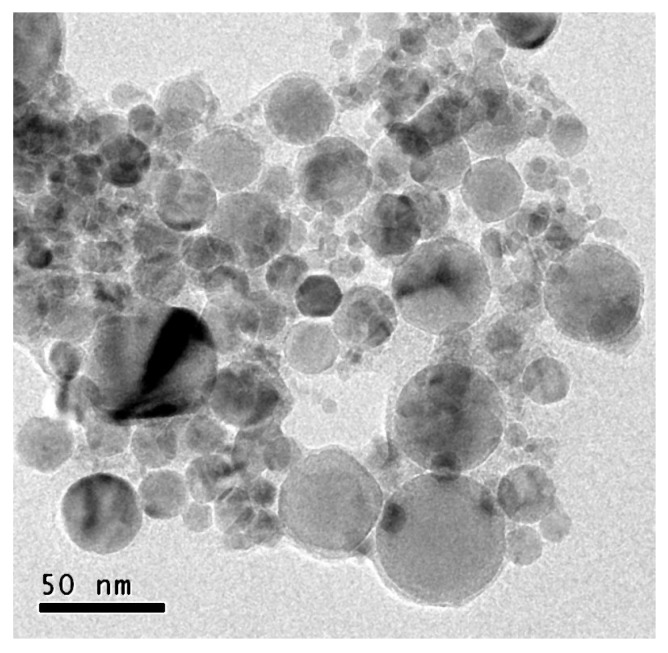
TEM image of a sample of TiO_2_ nanoparticles.

The nanofluids are prepared with a natural ester derived from sunflower seeds. Its main thermal and dielectric properties, provided by its manufacturer, are shown in Table 1. This includes those properties that condition each fluid’s cooling performance, such as density, viscosity and thermal conductivity; and those that represent quality regarding behavior as an electrical insulator, such as AC breakdown voltage and dissipation factor. More precisely, this last parameter represents how far a real isolator is from being ideal (tan δ = 0). High-purity spherical nanoparticles of titania, with a mean size between 10 and 20 nm in diameter, are used as the solid fractions of the nanofluids. TEM images of the nanoparticles confirmed this size distribution (Figure 1).

The preparation of the samples follows a two-step method. A certain amount of the nanoparticles is added to the natural ester, depending on the desired concentration, and then the mixture is set under magnetic stirring for 15 min, and followed by 12 h under ultrasonication. Thus, a homogeneous dispersion is obtained. Another 12 h pass to dissipate the bubbles before any measurement.

The nanofluids to be tested are prepared with concentrations of 0.1, 0.2, 0.5, 0.7 and 1 kg/m^3^, to ensure the finding of the optimal concentration. Additionally, a sample of base fluid, named 0 kg/m^3^, is subjected to the same preparation method to allow their comparison. Once the samples are prepared, they are tested following the methods set in the standards, when available. The properties on which the dielectric and thermal performances of a fluid depend in a transformer are measured. Each property characterization is done twice, with different samples of each fluid, following the methods set in the standards when available, and the results are averaged. A validation condition has been set for the experimental tests to ensure their repeatability, assuming a maximal 3% variation among the results of the two series of tests for each property respecting the mean value, except for the BDV measurements, as traditionally a larger tolerance is accepted [30].

The thermal conductivity is measured at different temperatures using a KD2 Pro Thermal properties analyzer (0.01 W/m·K accuracy). Based on the hot transient wire technique, it links the changes in electrical parameters of its inner circuit to the variation of temperature due to thermal conduction across the sample. From these data the thermal conductivity is obtained. Every 15 min the analyzer takes a measurement while samples cool down from 323 K to ambient temperature inside an oven. To determine the viscosity of the fluid samples, a rotatory viscosimeter Haake viscotester 550 (inaccuracy 0.5% of reading) is used. It measures the dynamic viscosity at different temperatures, controlled by an external fluid circuit linked to a thermostatic bath, by translating the opposition of the test fluid (braking torque) against the rotational movement of the viscometer shaft. Regarding the density evolution with temperature, it is estimated with a density meter Mettler Toledo DM40 (0.1 kg/m^3^ measurement error), at six different temperatures from 293 to 343 K. This device weights the test samples, which are introduced at a controlled volume and temperature, and thus obtains their densities.

Different dielectric properties are also measured according the methodologies set in the respective international standards by the International Electrotechnical Commission (IEC). The commercial devices used are specifically designed for these tests, and apply the corresponding standards, pre-loaded in their software. The AC breakdown voltage is determined by a BAUR DPA 75 dielectric oil tester (1 kV accuracy), using 400 mL of sample at rated temperature, according to IEC 60156 standard methodology [31]. Each sample is subjected to six successive breakdown tests, delayed one each other by one minute. The samples are placed in a vessel with semispherical electrodes separated by 2.5 mm. A BAUR DTL 2a is used to measure the resistivity (3% of reading uncertainty) and the loss factor (1% of reading uncertainty). These tests are carried out with 45 mL of sample at 363 K in a U-shaped vessel, as set in the IEC 60247 standard [32]. The intensity and power factor, respectively, of DC and AC currents across the test samples generated by the device, are translated into the magnitude of these two measured properties. The moisture content is also controlled, since the dielectric properties depend on this parameter, by Karl–Fischer titration (IEC 60814 standard [33]) in a Metrohm 899 coulombmeter. This device detects electrically the consumption of the water present in an oil sample and estimates its concentration in function of the reagents consumed during the reaction and the sample weight.

The Table 2 shows the measurement range of the different testers used, together with the measurement precisions given by their manufacturers or obtained from the specifications.

**Figure 2:**
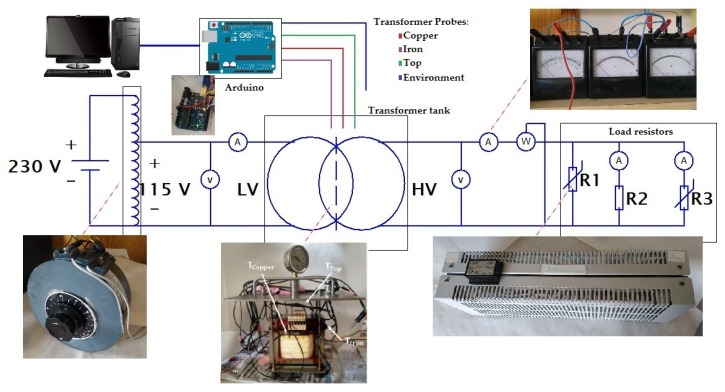
Connection scheme of the experimental platform.

**Figure 3:**
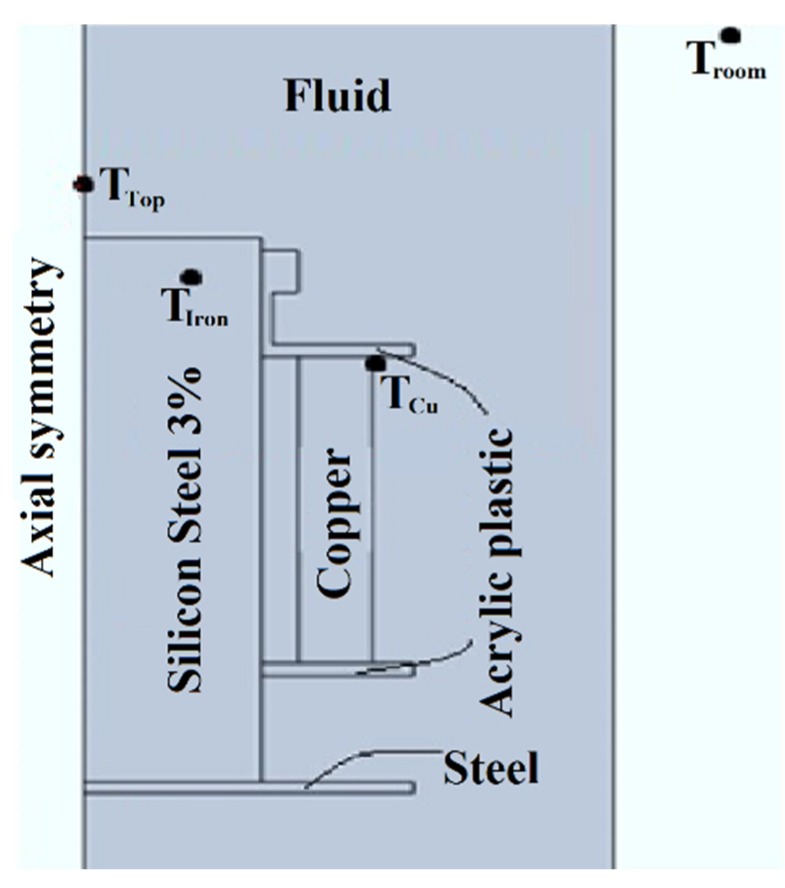
Section of the experimental transformer in the setup.

Following the experiment used by Patel et al. [34], an experimental setup was developed to study the cooling performance. The most promising nanofluid, determined according to the characterization of properties, and the base oil are tested in a prototype distribution transformer, comparing their cooling performances. This experimental platform is made with a small single-phase transformer (800 VA, 115/230V), shown in Figure 2, immersed in a tank. The two samples (about 6.5 L each) are poured in the 20 cm × 20 cm × 20 cm casing, made of stainless steel, until the transformer is completely covered with coolant. This design comes from experience gained in previous research [35]. The sample movement inside the tank is driven only by natural convection cycles that dissipate the heat produced during operation. The platform temperatures are monitored by three probes located in strategic places of the transformer (top, iron and windings). Figure 3 shows the approximate locations of the probes. Additionally, an external probe is used to measure the ambient temperature—the reference. The measurement and recording of the temperatures are made by a microcontroller (Arduino) and an Integrated Development Environment (IDE).

The platform was subjected to three tests with different load levels, represented by load indexes C (C = 0.7, C = 1, C = 1.3), depending on the power consumed by the circuit respecting its rated power. The load levels are reached by controlling three variable resistors, shown in the scheme of Figure 2. Table 3 summarizes the currents, voltages and resistances applied during the tests for each load index. Using these values, we assure the study of the behaviors of the samples at different temperatures. The overload regime gives also temperatures closer to those found in actual distribution and power transformers.

The temperatures of the probes are caught every five seconds during the tests until the steady-state is reached, and they are available during the process. The stability criteria followed are those defined in the IEC 60076-2, which establish that this condition is reached when the variation of the top oil’s temperature rises 1K⋅h^−1^ over a consecutive period of 3 h.

## 3. Results

### 3.1. Characterization of Properties

#### 3.1.1. Breakdown Voltage and Moisture Content

**Figure 4:**
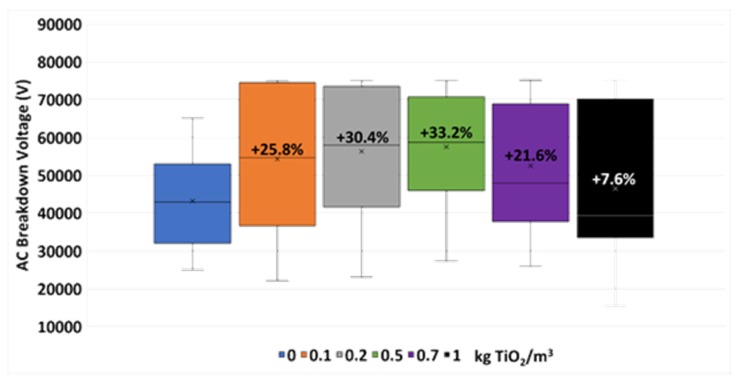
Breakdown voltage test results of the TiO_2_ nanofluids and the base fluid.

The results from the breakdown voltage tests are summarized in Figure 4. Here, the distribution values obtained during the tests are shown, including the mean values (represented by a cross) and the variations noticed in these mean values with respect to the one gotten with the base fluid, in percentages.

Here it is noticeable how the aforementioned variation increases with the nanoparticle concentration up to 33.2% with 0.5 kg/m^3^ of nanoparticles. From this sample the BDV starts to decrease progressively with the concentration (21.6% at 0.7 kg/m^3^ and 7.6% at 1 kg/m^3^). Thus, from the dielectric standpoint, 0.5 kg/m^3^ is the optimal concentration. This tendency is usually found in similar studies, including the one carried out with the same base fluid and Fe_2_O_3_ nanoparticles [29].

**Figure 5:**
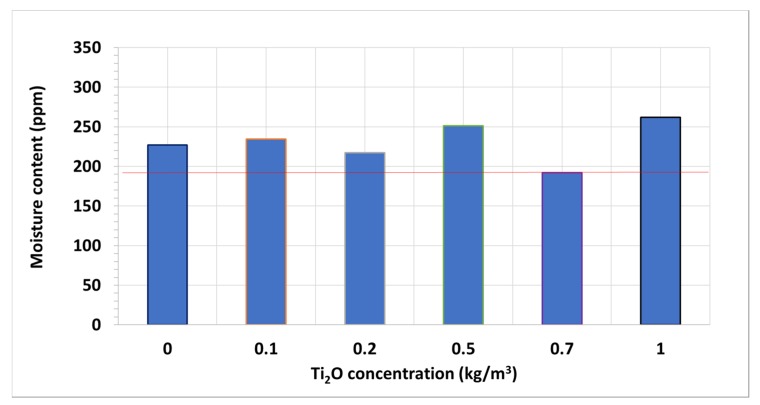
Mean moisture content of tested TiO_2_ nanofluid samples.

This occurs even though most of the nanofluid samples have slightly larger contents of moisture than the base fluid one, 226.9 ppm (Figure 5). These nanofluid samples may have shown improvements that were even more pronounced regarding the base fluid if they had the same moisture contents, according to the literature [36,37]. Additionally, the sample with the optimal concentration is also the second one with the most moisture (251.0 ppm), while the closer-concentration samples present moisture contents lesser than that of the base fluid (217.0 ppm in 0.2 kg/m^3^ and 191.9 ppm in 0.7 kg/m^3^ nanofluids). Thus, the optimal-concentration nanofluid may have been more noticeable if all the samples had the same moisture contents. Regarding the 1 kg/m^3^ nanofluid sample, although it presents the most moisture (261.9 ppm), this is no really far from some of the preceding samples. Therefore, the fall in breakdown voltage regarding the other nanofluids is not only attributable to the moisture, it is also due to an excessive concentration of nanoparticles, following the trend started at 0.7 kg/m^3^.

#### 3.1.2. Resistivity and Dielectric Dissipation Factor

**Figure 6:**
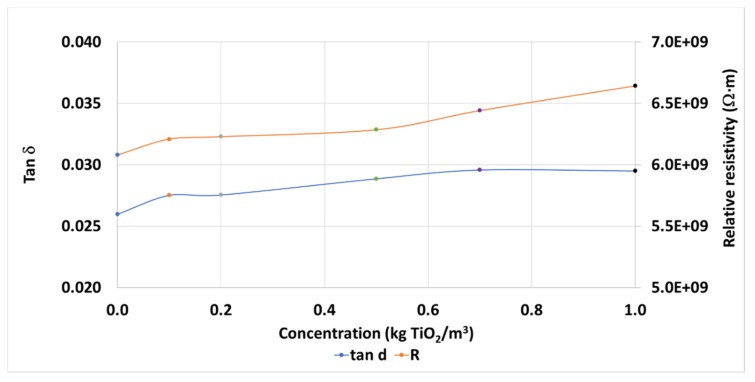
Mean dissipation factors and relative resistivities of TiO_2_ nanofluid samples.

Regarding other typical dielectric parameters measured in this kind of study, the Dielectric Dissipation Factor (tan δ) is affected by the presence of nanoparticles, and it is slightly increased, from 0.0260 to 0.0295, up 13.5%, depending on the concentration (Figure 6). On this occasion the effect is less pronounced than that seen with the Fe_2_O_3_ nanoparticles with the same base fluid, up to 119.7% in the concentrations studied [29]. This difference is also noticed when focusing on the resistivity of the samples, with a slight increase with the concentration of nanoparticles, from 6.081 GΩ·m to 6.642 GΩ·m, up to 9.2% (Figure 6) against that seen with the ferrofluid [29], whose resistivity decreased with the nanoparticles’ concentration, up to −31.4%.

#### 3.1.3. Thermal Conductivity

**Figure 7:**
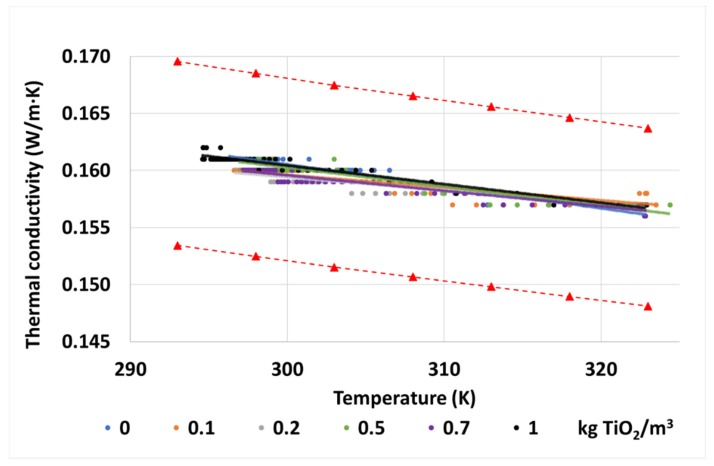
Thermal conductivities of the tested samples of base fluid and TiO_2_ nanofluids, including the uncertainty gap of the experiments according to the estimated error of the tester (red dashed lines).

As can be seen in Figure 7, as it happened with the maghemite nanoparticles [29], there is not a substantial variation of this property in the nanofluids regarding the base fluid. Here, the thermal conductivities obtained during the tests at different temperatures are represented, together with the trendlines obtained from them, and the estimated error gap of the test obtained from the conductivities of the base fluid. The conductivity values of the nanofluids are in the estimated error gap of the tester, represented by the red dashed lines, as variations of k are under 1% at any of the concentrations and temperatures studied, and all the tendency lines almost overlap. In view of these results, it can be pointed out that the thermal conductivity is not affected by the addition of these nanoparticles at the proposed concentrations.

In any case, according to the data provided by the oil manufacturer for the base fluid, the evolution of this property with the temperature is as expected. Due to the scarce effect of the nanoparticles, the nanofluids also follow the same tendency.

#### 3.1.4. Dynamic Viscosity and Density

To finish with the characterization of properties, the viscosities and densities of the samples were tested, as the performance of the natural convection cycles is conditioned by them.

**Figure 8:**
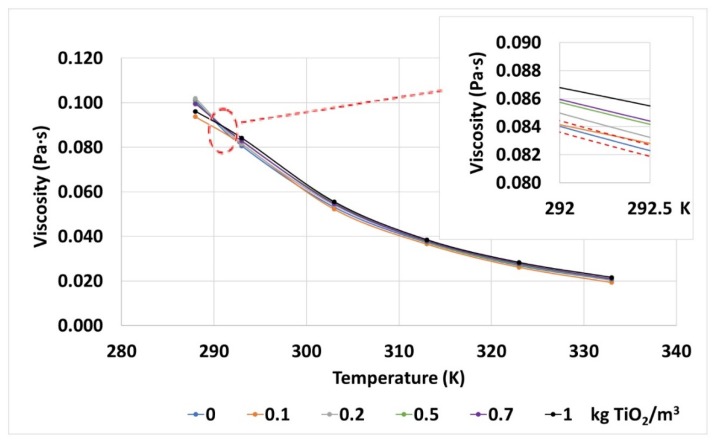
Dynamic viscosities measured for the base fluid and TiO_2_ nanofluids at different temperatures. The error gaps of the measurements are also shown by red dashed lines in the inset, top-right corner.

The viscosity seems to be affected by the addition of nanoparticles, as the mean variations found are higher than the test uncertainty, represented by the red dashed lines in Figure 8. These variations are up to 7.2%, depending on the nanoparticle concentration and on the temperature. Regarding these parameters, the influence of the nanoparticles on the viscosity is more noticeable as their concentration increases. Additionally, the effect of the temperature on this property is in line with the tendency of the base fluid in this sense, and the nanofluids still fulfill the recommendations set in the standards for vegetal dielectric oils (<0.0455 Pa·s at 313 K).

**Figure 9:**
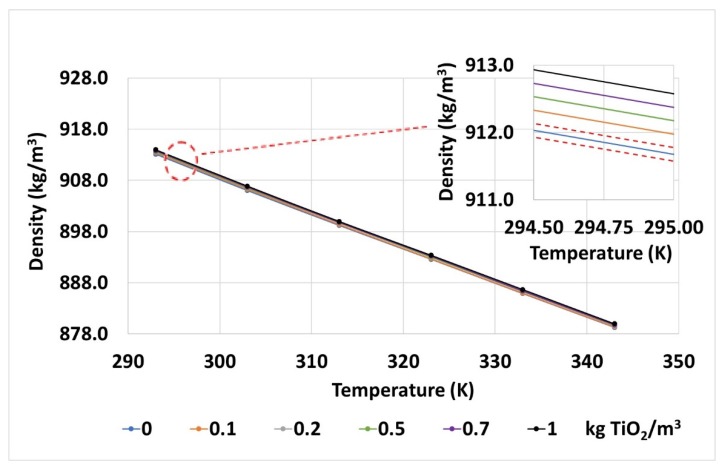
Densities measured for the base fluid and TiO_2_ nanofluids at different temperatures. The error gaps of the measurements are also shown by red dashed lines in the inset, top-right corner.

The density for its part shows a similar situation (Figure 9), a logical evolution with temperature, as expected, and with the nanoparticle concentration, it obtains mean values very close to those calculated with the quantities of solid fraction and base fluid used during the preparation (despicable punctual differences that are under test error).

### 3.2. Nanofluid Cooling Tests 

According to the former, the results of the characterization of the nanofluids and the base fluid reflect an important improvement of the dielectric properties. On the other hand, the addition of these nanoparticles has a negligible effect on the thermal properties that condition the cooling performance of a fluid, since the thermal conductivity can be considered constant and the viscosity and density suffer from limited variation. In view of this situation, it can be inferred that the presence of nanoparticles has a generally positive effect on the base fluid properties. Thus, the resulting optimal TiO_2_ nanoparticle concentration seems to be 0.5 kg/m^3^; that with the largest improvement of BDV of the base fluid, and that which shows a variation in viscosity of up to 3.5%—not the largest range experimentally determined. Samples of this optimal nanofluid and also the base fluid have been tested in an experimental setup, to compare their behaviors as cooling fluids, while we tried to check that the addition of the nanoparticles was not detrimental for the cooling performance of the oil, as it should be according to the characterization test results.

**Figure 10:**
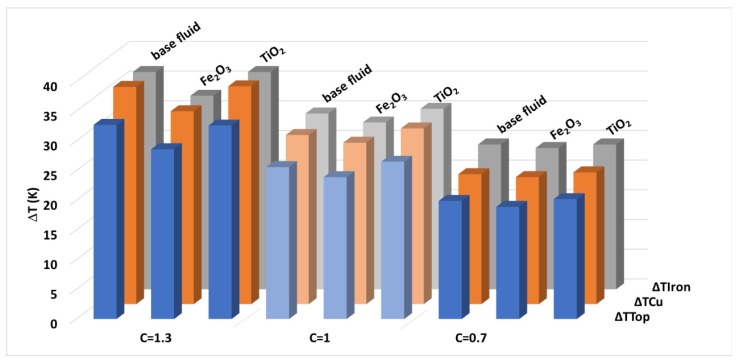
Temperature gradients found in each probe with different load conditions and cooling fluids.

The results of these tests, in the form of mean temperature gradients regarding the ambient temperature, registered by the equipment once the test stability condition was reached, are summarized in Table 4. The cooling of the platform with titania nanofluids is slightly worse in comparison with what occurs with the base fluid, since the gradients of most of the probes are higher (up to 3.9% more), for the three load levels. The different cooling behaviors of the titania nanofluids are even more noticeable when comparing the test results with those obtained for the ferrofluids [29]. In this last case, a clear improvement in the cooling of the transformer was found, especially at the largest load levels, gradients up to 12% lower (Figure 10).

## 4. Discussion

Among the great variety of characterization test results in this kind of research, some of these studies were carried out in similar conditions and also show similar results. 

For instance, regarding the BDV, Zhong et al. studied the breakdown voltages of different titania nanofluids with vegetal oils as base fluids [38]. They noticed an improvement of BDV close to 30%, around 0.3 g/L of nanoparticle concentration. Additionally similar results were obtained with titania in a mineral base [39]. According to the more spread theory [40,41], the capacity to improve this parameter is due to the capacity of nanoparticles to capture and to slow down the electrons rising during the ionization of oil under electric fields. These free electrons promote the development of streamers once launched, reducing the capacity of the dielectric oils to withstand voltages; that is, their isolating capacity. This effect might be lost at larger concentrations of nanoparticles, as it occurs in this case, since they build conductive bridges that ease the flow of electrons [20].

On the other hand, dielectric dissipation factor (tan δ) or resistivity vary according to the tendencies herein exposed, in opposite ways to each other in fact, with the nanoparticles’ concentration [42,43,44], depending on their conductive nature. The presence of nanoparticles affects the polarizability and the electrical conductivity of the base fluid, and consequently these properties. In this case it is remarkable how the affectation of the loss factor with the semi-conductive nanoparticles (TiO_2_) is several times lower than that seen with a conductive nanoparticle, although its concentration was more than doubled [29]. According to this, the shorter variations of resistivity and dissipation factor are produced with the same kind of nanoparticle—those with a semi-conductive nature (TiO_2_). This has already been seen in other works with nanoparticles of a different nature [45,46]. This also agrees with the previous BDV results, as the nanoparticle capable of a better enhancement of this parameter in the base fluid (TiO_2_) is the one that presents an improvement in its resistivity and a lower increase of the loss factor.

In the case of the thermal properties, the limited affectation found can be easily due to the low concentrations used in this research. Despite that the majority of references reflect improvements of the thermal conductivity beyond the uncertainty gap of this research, there are examples at similar concentrations with affectations close to 0, with titania nanoparticles [17] or other species [46,47], in mineral oils. The same could be said about the viscosity, as there exist studies with limited variations in this parameter due to the nanoparticles [40,41,47].

This situation agrees with the results of the cooling tests, as a slight increase in the temperature gradients measured when the TiO_2_ nanofluid is applied can be explained by the combination of a constant thermal conductivity and a limited increase of viscosity. When comparing these results with those obtained with maghemite nanofluids from a previous study [29], the situation is almost the same regarding the characterization of the thermal properties, as the thermal conductivity and viscosity also remained almost constant. Nevertheless, the results during the cooling of an experimental transformer were far from being the same, since the ferrofluid improves the cooling performance of the TiO_2_ nanofluid and of the base fluid at the setup.

The absence of an improvement of the cooling with the non-magnetic TiO_2_ nanoparticles, contrarily to their maghemite counterparts, can only be read as another sign that supports the presence of thermomagnetic buoyancy forces when the nanoparticles used are magnetic. That is even more true if the resulting nanofluids have proved to be quite similar during their characterization.

The presence of these additional buoyancy forces, proposed as an explanation in the previous study [29], has already been proposed and supported by other researchers [13,34,48].

## 5. Conclusions

The addition of TiO_2_ commercial nanoparticles to a vegetal commercial dielectric oil seems to fulfill the expected consequences on the properties of the base oil, with an improvement on its breakdown voltage of up to 33.2%. As with using Fe_2_O_3_ nanoparticles, an optimal concentration was noticeable, 0.5 kg/m^3^ in this case. As also occurred previously, the affectation with the studied concentrations of the thermal properties (thermal conductivity, viscosity or density) was scarce, even despicable.

This was confirmed when applying the optimal nanofluid to the cooling of an experimental transformer, as, on the contrary to the previous experience with Fe_2_O_3_ nanoparticles, the performance of the tested nanofluid was slightly worse than that with the base fluid (3.9% cooling worsening with TiO_2_ nanofluid vs. 12% improvement with Fe_2_O_3_ nanofluid, regarding the base fluid’s performance). The different behaviors among the TiO_2_ and Fe_2_O_3_ nanofluids at the experimental setup, together with their similarities regarding their properties characterization, seem to support the explanation given for the better performance of the maghemite nanofluid: the existence of thermomagnetic buoyancy forces, considering the non-magnetic nature of the titania nanoparticles.

## Figures and Tables

**Table 1:** Main properties of the vegetal base fluid.

**Density (283 K) (kg/m^3^)**	910
**Dynamic viscosity (313 K)(Pa·s)**	<0.0455
**Thermal conductivity (298 K)(W/K·m)**	0.1691
**Dissipation factor (Tan δ)**	<0.05
**AC Breakdown Voltage (kV)**	>35

**Table 2:** Measurement range and precision of the testers employed during the research.

Tester	Property	Measurement Range	Measurement Precision
**Baur DPA 75**	Breakdown voltage	0–75 kV	1 kV
**Baur DTL 2a**	Tan δ	0.00001–4.910	0.0001–0.0491
	Resistivity	2.5 MΩ·m–20 TΩ·m	0.075 MΩ·m–0.6 TΩ·m
**KD2 pro**	Thermal Conductivity	0.02–0.2 W/m·K	0.01 W/m·K
**Haake 550**	Dynamic viscosity	0.02–7 Pa·s	0.0001–0.035 Pa·s

**Table 3:** Circuit parameters during the load tests.

**Overload C = 1.3; P = 1040 W**	
Primary winding	Secondary winding
I_p_ = 9 A V_p_ = 115 V	I_s_ = 4.5 A V_s_ = 230 V R_eq.s_ = 51.11 Ω
**Rated power C = 1; P = 800 W**	
Primary winding	Secondary winding
I_p_ = 6.96 A V_p_ = 115 V	I_s_ = 3.48 A V_s_ = 230 V R_eq.s_ = 65.71 Ω
**Underload C = 0.7; P = 560 W**	
Primary winding	Secondary winding
I_p_ = 4.87 A V_p_ = 115 V	I_s_ = 2.43 A V_s_ = 230 V R_eq.s_ = 94.46 Ω

**Table 4:** Mean experimental gradients of temperature (K) for each fluid, load index C and probe.

C	Fluid	ΔT_Top_	ΔT_Cu_	ΔT_Iron_
1.3	Base fluid	32.7	36.6	36.6
	0.5 kg/m^3^ Nanofluid	32.6	36.7	36.6
1	Base fluid	25.6	28.5	29.6
	0.5 kg/m^3^ Nanofluid	26.5	29.6	30.4
0.7	Base fluid	19.9	21.9	24.4
	0.5 kg/m^3^ Nanofluid	20.2	22.2	24.4
